# Deletion of NFIA Leads to Activation of S100A7 and Inflammatory Response‐Induced Apoptosis of Keratinocytes in Oral Lichen Planus Progression

**DOI:** 10.1002/kjm2.70052

**Published:** 2025-06-25

**Authors:** Maimaiti Tudi, Zumulaiti Aierken, Maihebubaimu Tuerxun, Maimaitituxun Tuerdi

**Affiliations:** ^1^ Department of Stomatology The First People's Hospital of Kashi Kashi Xinjiang People's Republic of China; ^2^ Department of Oral and Maxillofacial Trauma and Orthognathic Surgery The First Affiliated Hospital of Xinjiang Medical University (Affiliated Stomatological Hospital) Urumqi Xinjiang People's Republic of China

**Keywords:** inflammatory response, keratinocyte, NFIA, oral lichen planus, S100A7

## Abstract

S100 calcium‐binding protein A7 (S100A7) has been implicated in psoriasis and other inflammatory diseases. However, the function of S100A7 in oral lichen planus (OLP), a chronic inflammatory disease, remains unclear. OLP was induced in mice by transplanting human OLP lesions into the backs of thymus‐free mice, and an in vitro cell model was constructed using LPS‐stimulated HaCaT cells. Gene intervention was performed using shRNA lentiviral vectors. The secretion of the pro‐inflammatory factors IL‐6 and TNF‐α, as well as the rate of apoptosis in HaCaT cells, was also assessed. ChIP assay and dual‐luciferase assay were used to validate the transcriptional regulation of S100A7 by nuclear factor 1 A‐type (NFIA). The expression of S100A7 was significantly elevated in the lesion tissues of OLP‐induced mice. Knockdown of S100A7 alleviated inflammation and reduced keratinocyte apoptosis. The transcription factor NFIA repressed S100A7 expression by binding to the S100A7 promoter. The overexpression of NFIA ameliorated inflammation in vivo and reduced apoptosis in vitro, which was abrogated by further overexpression of S100A7. Overall, our results indicate that NFIA reduces inflammatory response‐induced keratinocyte apoptosis in OLP by inhibiting S100A7 transcription.

## 1 Introduction

Oral lichen planus (OLP) is a chronic or recurrent inflammatory autoimmune disease of the oral mucosa characterized by white lace‐like lesions, with or without atrophic or erosive areas [1]. Although the etiology remains unknown, antigen‐specific mechanisms of OLP include antigen presentation by basal keratinocytes and keratinocyte killing by CD8^+^ T cells [2]. Although OLP is a benign disorder, 1.4% of oral cavity lesions undergo malignant transformation within 7 years [3]. Topical super‐potent corticosteroids constitute the first‐line treatment for symptomatic flares, whereas available second/third‐line treatments include topical calcineurin inhibitors, systemic corticosteroids, systemic retinoids, and topical/systemic immunomodulators [4]. However, approximately 20% of the patients receiving intermittent topical glucocorticoid treatment for OLP had glucocorticoid‐induced adrenal insufficiency [5]. Therefore, the exploration of the molecular mechanisms involved might be helpful for the development of new targets in the field of OLP.

S100 proteins are calcium‐binding proteins that are expressed only in vertebrates and interact with enzymes, receptors, transcription factors, and nucleic acids to modulate proliferation, differentiation, apoptosis, and inflammation [6]. Here, we identified S100 calcium‐binding protein A7 (S100A7, also called psoriasin) as the most significantly expressed gene in oral mucous biopsies and the OLP epithelium of patients with OLP, which was thus selected as the target of our interest. Interestingly, S100A7 expression is induced by Toll‐like receptor 3 to control keratinocyte differentiation following skin injury [7]. Keratinocytes, the main cells of the epidermis, are the first‐line of defense against many viruses, and their counterparts S100A8 and S100A9 are both expressed at low levels in the normal epidermis, which is highly induced in inflammatory skin diseases such as psoriasis, OLP, and lupus erythematosus [8]. TNF‐α levels were significantly higher in both erosive and reticular OLP patients than in the healthy group, and certain salivary cytokines, such as IL‐6, were positively correlated with OLP severity [9]. Therefore, we anticipate that S100A7 is an important regulator of keratinocyte inflammation in OLP. Nuclear factor one (NFI) site‐specific DNA‐binding proteins constitute a group of transcription factors that are imperative for the development of multiple organ systems [10]. All four family members, NFIA, NFIB, NFIC, and NFIX, have a homologous DNA‐binding domain and can modulate cell proliferation and differentiation via transcriptional control of their targets [11]. Although we predicted NFIA to be a transcription factor responsible for the overexpression of S100A7 in keratinocytes, the molecular mechanisms have yet to be clearly explained. To better understand this, we explored the mechanism by which NFIA/S100A7 signaling regulates the keratinocyte inflammatory response and apoptosis in OLP.

## 2 Methods and Materials

### 2.1 Sample Collections

The study protocol was approved by the Ethics Committee of the First People's Hospital of Kashi. All experiments involving human samples were conducted following the Declaration of Helsinki. Informed consent was obtained from all participants before the study. The diseased and adjacent normal mucosa of 14 patients with OLP were collected and subjected to histopathological examination. There were 8 cases of reticular, 2 cases of erosive, and 4 cases of papular subtype (Supporting Information). The reticular samples were immediately transplanted into Eagle's minimum essential medium (M0628; Sigma‐Aldrich Chemical Company; St Louis, MO, USA) to prepare explants approximately 1 cm in diameter for grafting [12].

### 2.2 Animal Studies

The Institutional Ethical Committee of the First People's Hospital of Kashi approved all animal studies following ARRIVE guidelines. Thymus‐free mice (NU/J, 002019, Jackson Laboratory; Sacramento, CA, USA) were housed in a single‐layer sterile plastic film isolation system (Global Biotech; Suzhou, Jiangsu, China) and provided with sterile water, feed, and bedding. Overexpression lentiviruses of NFIA (OE‐NFIA) and S100A7 (OE‐S100A7), knockdown lentivirus of S100A7 (sh‐S100A7), and their control lentiviruses (OE‐NC, sh‐NC) (titer: > 10^9^ TU/mL) were purchased from VectorBuilder (Guangzhou, Guangdong, China). Mice were genetically intervened using lentiviral vectors via syringe injection into the dorsal portion of the inoculated grafts.

For mouse OLP modeling, 1 day before lentiviral vector injection, the mice were anesthetized with 100 mg/kg ketamine and 5 mg/kg valium (i.p.). The skin on the backs of the mice was wiped with 70% ethanol to remove two flaps approximately 1 cm in diameter, which were replaced with grafts and covered with a sterile bandage. The mice were placed in an isolator, and the bandages were removed after 2 weeks. The mice were euthanized after 6 weeks, and the grafts were removed for subsequent experiments [12].

### 2.3 Cell Culture and Treatment

HaCaT cells (BNCC339817, BeNa Culture Collection, Beijing, China) were placed in an incubator at 37°C with a CO_2_ concentration of 5% and cultured in 90% DMEM‐H and 10% FBS (BNCC338068, BeNa). To mimic the local immune microenvironment of OLP, the medium was replaced with keratinocyte serum‐free medium (KSFM) (17,005,042, Thermo Fisher), in which HaCaT cells were cultured overnight and then stimulated for 48 h with LPS (HY‐D1056, MedChemExpress) at a concentration of 10 μg/mL [13]. The control group was treated with equal amounts of DMSO.

Cells were seeded in 96‐well plates at 37°C in a humidified atmosphere containing 5% CO_2_. On the second day, the overexpression lentiviral vector (> 10^9^ TU/ml, VectorBuilder) for S100A7 and NFIA human genes, S100A7 human gene shRNA lentiviral vector (> 10^9^ TU/mL, VectorBuilder), and pCMV6‐A‐Puro empty vector (PS100025, OriGene Technologies; Beijing, China) were used to infect cells at 37°C. The infected medium was replaced with 2 mL of fresh medium. The infected cells were allowed to grow in the medium for 2–3 days and were screened with a puromycin‐containing medium (T19978, TargetMol, Shanghai, China). After screening, the cells were stimulated with LPS for 48 h.

### 2.4 Reverse Transcription‐Quantitative PCR (RT‐qPCR)

Total RNA from OLP lesion tissues and HaCaT cells was extracted using the TRIzol reagent (R0016, Beyotime Biotechnology Co. Ltd., Shanghai, China). cDNA was reverse‐transcribed from total RNA using the TransScript RT kit (AT101‐02, Transgen; Beijing, China) and TB Green Premix Ex Taq (RR820Q, Takara Holdings Inc., Kyoto, Japan). qPCR was performed in a TSQ‐4048 fluorescence qPCR instrument (TSQ‐4048, Transgen), and the reaction conditions were applied, which were 95°C for 5 min, followed by 40 cycles of 95°C for 15 s and 60°C for 1 min. GAPDH was used as an internal control, and the relative expression was assessed using the comparative CT method (2^−ΔΔCT^). The primer sequences were as follows: S100A7 human (5′‐AGAAGCCAAGCCTGCTGACGAT‐3′ and 5′‐GTCCTTTTTCTCAAAGACATCGGC‐3′); NFIA human (5′‐GTGGAGGATGAAATGGACAGTCC‐3′ and 5′‐CTGCTGAAACCAGACTTCTCCG‐3′); GAPDH human (5′‐GTCTCCTCTGACTTCAACAGCG‐3′ and 5′‐ACCACCCTGTTGCTGTAGCCAA‐3′).

### 2.5 Enzyme‐Linked Immunosorbent Assay (ELISA)

HaCaT cells were seeded in 96‐well plates (5 × 10^4^ cells/well) and cultured in KSFM for 24 h. After treatment with LPS, the cell culture supernatant was collected to measure the concentrations of the pro‐inflammatory factors interleukin (IL)‐6 (E‐EL‐H6156, Elabscience Biotechnology Co. Ltd., Wuhan, Hubei, China) and tumor necrosis factor (TNF)‐α (E‐EL‐H0109, Elabscience) using the corresponding ELSA kit according to the manufacturer's instructions.

The treated OLP lesion tissues were rinsed with pre‐cooled PBS (0.01 M, pH 7.4, P4474, Sigma‐Aldrich) to remove residual blood, and the tissues were weighed and cut into pieces. After adding the clipped tissues to a glass homogenizer with a corresponding volume of PBS (tissue weight: PBS volume = 1:9), they were ground on ice. The homogenate was sonicated and centrifuged at 8°C, 5000 × g for 5–10 min, and the supernatant was collected to measure the concentrations of pro‐inflammatory factors IL‐6 and TNF‐α using the corresponding ELISA kits.

### 2.6 Western Blot

Cells or clinical mucosal tissues were lysed with RIPA buffer (P0013B, Beyotime) containing a mixture of phosphatase inhibitors (P1081, Beyotime) and phenylmethylsulfonyl fluoride (ST505, Beyotime). Proteins were separated by 10% sodium dodecyl sulfate‐polyacrylamide gel electrophoresis and electroblotted onto PVDF membranes. PVDF membranes were sealed with 5% skimmed milk for 1 h at room temperature and incubated overnight at 4°C with the appropriate primary antibodies against S100A7 (1:1000, RQ4106, NSJ Bioreagents; San Diego, CA, China), NFIA (1:1000, A3258, ABclonal; Wuhan, Hubei, China), IL‐6 (1:1000, A0286, Abclonal), TNF‐α (1:1000, ab183218, Abcam Inc., Cambridge, UK), P53 (1:1000, ab33889, Abcam), BAX (1:1000, ab32503, Abcam), and GAPDH (1:500, NB100‐56875, Novus Biological Inc., Littleton, CO, USA). The secondary antibody was HRP‐conjugated goat anti‐rabbit IgG (H+L) (1:10000, 31,460, Thermo Fisher). Positive signals were detected using BeyoECL Plus (P0018S; Beyotime).

### 2.7 TUNEL Assay

HaCaT cells were fixed in 4% paraformaldehyde (P1110, Solarbio) for 30 min and then incubated in a 0.3% hydrogen peroxide solution prepared in PBS for 20 min at room temperature. Staining was performed using the TUNEL FITC Apoptosis Detection Kit (A111‐01; NanJing Vazyme Biotech Co. Ltd., Nanjing, China). Cell nuclei were stained with DAPI (D9542, Sigma‐Aldrich). Observations were performed using a fluorescence microscope.

### 2.8 Luciferase Assay

The luciferase reporter plasmid was constructed by inserting the promoter fragment of S100A7 into the luciferase reporter vector of pGL3 Luciferase Reporter Vectors (E1751, Promega Corporation; Madison, WI, USA) using restriction endonucleases. The luciferase reporter gene plasmid was then transfected into HaCaT cells overexpressing NFIA using Lipofectamine 2000 (11,668,500, Thermo Fisher Scientific) and cultured for 36 h. Luciferase activity was measured using the Dual‐Luciferase Reporter Gene Assay System (E1910; Promega).

### 2.9 Chromatin Immunoprecipitation (ChIP) Assays

ChIP analysis was performed using a ChIP commercial kit (17–295, Sigma‐Aldrich). The cells were then washed with cold PBS, cross‐linked with 1% formaldehyde, and treated with 0.125 M glycine. The cells were sonicated in ChIP lysis buffer to generate a 600 bp fragment, followed by overnight incubation at 4°C with antibodies against NFIA (1:50, 69375S, Cell Signaling Technologies; Beverly, MA, USA) and Rabbit IgG, polyclonal Isotype Control (1:1000, ab171870, Abcam). After precipitation of chromatin‐antibody complexes with protein‐A agarose beads, the proteins were disassembled and removed with proteinase K (26,160, Thermo Fisher). qPCR was performed to analyze S100A7 promoter enrichment.

### 2.10 Statistical Analysis

All the experimental data were obtained from at least three independent replicates. All data are presented as mean ± standard error of the mean, and a paired or unpaired *t*‐test was used to assess significant differences between any two groups. The significance among multiple groups was determined by ANOVA with GraphPad Prism 8.0.6 (San Diego, CA, USA). For all statistical tests, statistical significance was set at *p* < 0.05.

## 3 Results

### 3.1 Elevated S100A7 Expression Is Identified in OLP Lesion Tissues

**FIGURE 1:**
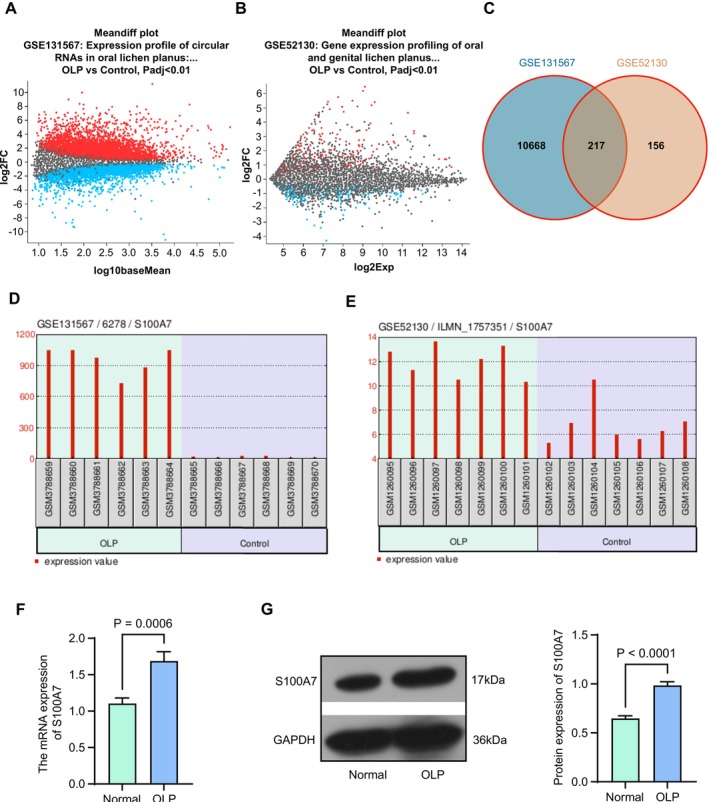
Higher expression of S100A7 is identified in lesion tissues of OLP patients. (a) The volcano map of differentially expressed genes in the GSE131567 dataset. (b) The volcano map of differentially expressed genes in the GSE52130 dataset. (c) The intersection of differentially expressed genes in both datasets. (d) The expression pattern of S100A7 in the GSE131567 dataset. (e) The expression pattern of S100A7 in the GSE52130 dataset. (f) The mRNA expression of S100A7 in OLP lesion tissues as well as normal tissues was examined using RT‐qPCR (*n* = 14). (g) The positive rate of S100A7 in OLP lesion tissues as well as normal tissues was examined using western blot analysis (*n* = 14). *p* values were analyzed by paired *t*‐tests. Data are the means ± SEM.

We analyzed differentially expressed genes between the six oral mucous biopsies from untreated patients with OLP (GSM3788659–GSM3788664) and six normal oral mucous tissues from healthy patients (GSM3788665–GSM3788670) in the GSE131567 dataset (Figure 1A) and between the OLP epithelium (GSM1260095–GSM1260101) and the normal oral epithelium (GSM1260102–GSM1260108) in the GSE52130 dataset (Figure 1B). *p* value correction was conducted using Benjamini and Hochberg, and the screening threshold was *p* adj < 0.01. The intersection of the differentially expressed genes screened by the two was taken, and the results showed that there were 217 intersecting genes (Figure 1C). Further analysis of the expression of intersecting genes in the OLP showed that S100A7 (*p* adj = 4.99E‐232) and RNF165 (*p* adj = 0.000125) were the most significantly differentially expressed genes in the GSE131567 and GSE52130 datasets, respectively. S100A7 was upregulated in the oral mucosa or epithelial tissues of OLP patients in both GSE131567 (Figure 1D) and GSE52130 datasets (LogFC > 0) (Figure 1E).

First, we obtained tissue samples from diseased and normal mucosa of 14 patients with OLP and detected significantly higher mRNA expression of S100A7 in OLP lesion tissues than in normal tissues by RT‐qPCR (Figure 1F). Western blot analysis of OLP lesion tissues revealed that the protein expression of S100A7 in OLP was higher than that in the normal group (Figure 1G).

### 3.2 Knockdown of S100A7 Inhibits Pro‐Inflammatory Cytokine Secretion and Apoptosis in LPS‐Treated HaCaT Cells

**FIGURE 2:**
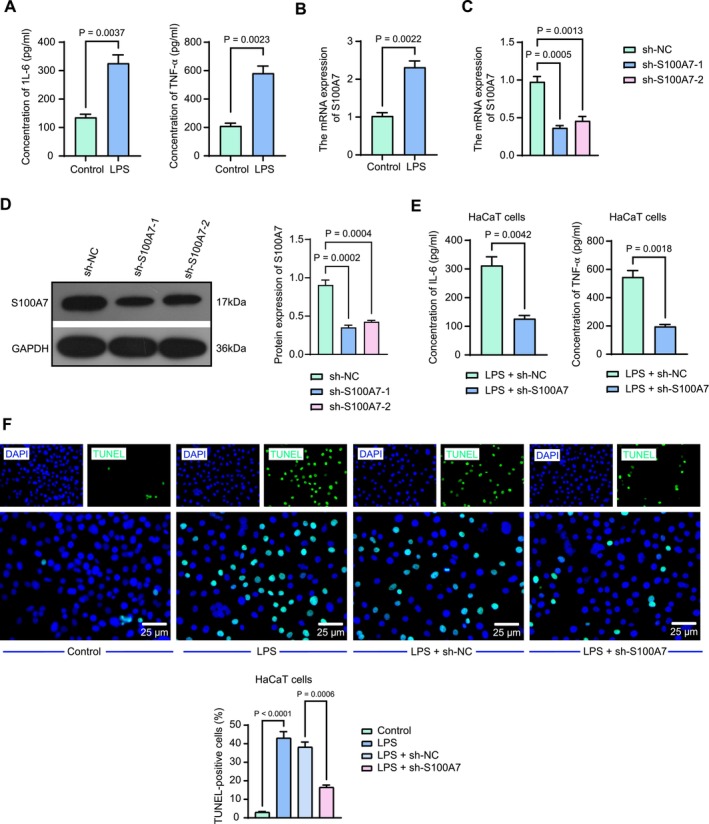
Knockdown of S100A7 inhibits pro‐inflammatory cytokine secretion and apoptosis in LPS‐treated HaCaT cells. (a) IL‐6 and TNF‐α levels in HaCaT cells induced with LPS or not were examined using ELISA. (b) S100A7 mRNA expression level in LPS‐treated HaCaT cells was examined using RT‐qPCR. (c) shRNA knockdown efficiency in HaCaT cells was examined using RT‐qPCR. (d) Validation of sh‐S100A7 knockdown efficiency in HaCaT cells by Western blot analysis. (e) IL‐6 and TNF‐α levels in HaCaT cells infected with sh‐NC or sh‐S100A7 were examined using ELISA. (f) The representative images and TUNEL‐positive cells in HaCaT cells infected with sh‐NC or sh‐S100A7. *p* values were analyzed by unpaired *t*‐tests or one‐way ANOVA. Data are the means ± SEM and are representative of three independent experiments.

The immune microenvironment of OLP in vitro was mimicked by stimulating HaCaT cells with LPS (10 μg/mL) in KSFM for 48 h. A significant increase in the levels of IL‐6 and TNF‐α was detected using ELISA (Figure 2A). Enhanced S100A7 mRNA expression in LPS‐treated HaCaT cells was detected by RT‐qPCR (Figure 2B). Two shRNAs (shS100A7–1 and shS100A7–2) were designed and synthesized to infect HaCaT cells. The knockdown efficiency was verified by RT‐qPCR, and the results illustrated that the mRNA expression of S100A7 was reduced in lentivirally infected HaCaT cells (Figure 2C). The shS100A7–1 exhibiting a higher infection rate was confirmed by western blot analysis and selected for subsequent experiments (Figure 2D). The infected HaCaT cells were stimulated with LPS for 48 h. Determination of IL‐6 and TNF‐α levels in HaCaT cell culture supernatants using ELISA revealed that the secretion of IL‐6 and TNF‐α was significantly reduced after S100A7 knockdown (Figure 2E). Apoptosis in HaCaT cells was assessed using the TUNEL assay. The TUNEL‐positive cell rate was significantly higher after LPS induction, and the TUNEL‐positive cell rate in HaCaT cells with S100A7 knockdown was significantly lower (Figure 2F).

### 3.3 Knockdown of S100A7 Inhibits Inflammatory Response and Apoptosis in Lesion Tissues From Mice With OLP

**FIGURE 3:**
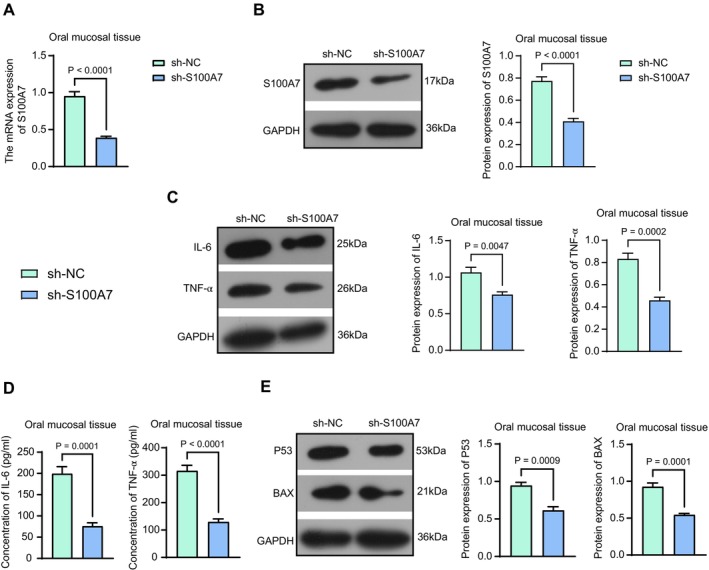
Knockdown of S100A7 inhibits inflammatory response and apoptosis in lesion tissues of mice with OLP. (a) shRNA knockdown efficiency in lesion tissues of mice treated with sh‐NC or sh‐S100A7 was examined using RT‐qPCR. (b) Validation of sh‐S100A7 knockdown efficiency in lesion tissues by Western blot analysis. (c) The protein expression of IL‐6 and TNF‐α in lesion tissues of mice treated with sh‐NC or sh‐S100A7 was examined using western blot analysis. (d) IL‐6 and TNF‐α levels in lesion tissues of mice treated with sh‐NC or sh‐S100A7 were examined using ELISA. (e) The protein expression of P53 and BAX in lesion tissues of mice treated with sh‐NC or sh‐S100A7 was examined using western blot analysis. *p* values were analyzed by unpaired t‐tests. Data are the means ± SEM (*n* = 5).

To validate the effect of S100A7 on OLP, we used thymus‐free mice and administered lentiviral vectors carrying sh‐S100A7 (shS100A7–1). The knockdown efficiency was verified by RT‐qPCR, and the mRNA expression of S100A7 was reduced in OLP lesion tissues after sh‐S100A7 infection (Figure 3A). The protein expression of S100A7 in OLP lesion tissues was analyzed by western blot analysis, which indicated that S100A7 protein expression was decreased in the sh‐S100A7 group compared with the sh‐NC group (Figure 3B). Protein expression and concentrations of pro‐inflammatory cytokines (IL‐6 and TNF‐α) in OLP lesion tissues after the knockdown of S100A7 were detected using western blot analysis and ELISA. A significant decrease in IL‐6 and TNF‐α protein expression and concentration was observed (Figure 3D,E). In addition, the protein expression of P53 and BAX was examined using western blot analysis, which was repressed after the knockdown of S100A7 (Figure 3E).

### 3.4 NFIA Acts as an Upstream Transcription Factor of S100A7 to Repress S100A7 Expression

**FIGURE 4:**
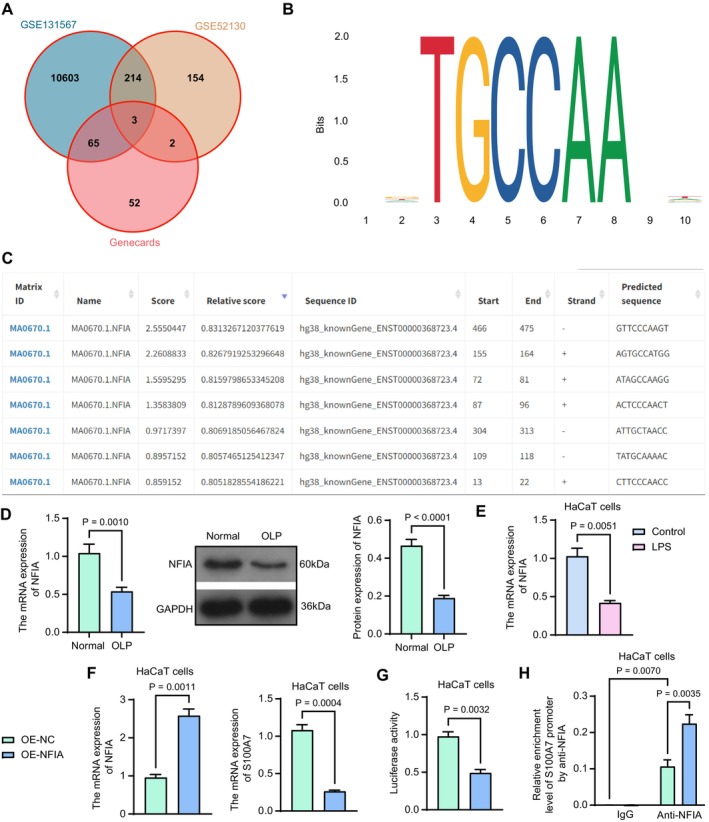
Transcription factor NFIA inhibits S100A7 expression in HaCaT cells. (a) The intersection of transcription factors binding to the S100A7 promoter and the differentially expressed genes in the GSE131567 and GSE52130 datasets. (b) The binding matrix of NFIA. (c) The binding sites between NFIA and the S100A7 promoter were predicted in the JASPAR database. (d) The expression of NFIA in OLP lesion tissues as well as normal tissues was examined using RT‐qPCR and western blot analysis (*n* = 14). (e) The mRNA expression of NFIA in HaCaT cells induced with LPS was examined using RT‐qPCR. (f) mRNA expression of NFIA and S100A7 in HaCaT cells infected with OE‐NC or OE‐NFIA was examined using RT‐qPCR. (g) The effect of the overexpression of NFIA on S100A7 luciferase activity was examined using a dual‐luciferase assay. (h) anti‐NFIA enrichment near the S100A7 promoter was examined using ChIP. *p* values were analyzed by unpaired *t*‐tests or two‐way ANOVA. Data are the means ± SEM and are representative of three independent experiments.

To explore the causative factors for the differential expression of S100A7 in OLP, we downloaded a list of transcription factors that have binding relationships near the S100A7 promoter from the GeneCards database (https://www.genecards.org/) and intersected them with the differentially expressed genes in the GSE131567 and GSE52130 datasets. There were three intersections: NFIA, cyclic AMP‐responsive element‐binding protein 1 (CREB1), and chromobox protein homolog 3 (CBX3) (Figure 4A). Among them, NFIA was the most significantly differentially expressed gene in these two datasets (GSE131567: *p* adj = 5.05E‐14, GSE52130: *p* adj = 0.002058). Moreover, NFIA expression was reduced in both the oral mucosa and epithelial tissues of patients with OLP (logFC < 0). In the Jaspar database (https://jaspar.elixir.no/), we found multiple binding sites (Figure 4B,C) between NFIA and the vicinity of the promoter of S100A7, suggesting that NFIA may inhibit chronic inflammation in OLP by regulating the transcriptional activity of S100A7.

Next, we investigated whether NFIA affects OLP through transcriptional regulation of S100A7. RT‐qPCR and western blot results showed that NFIA expression was significantly reduced in the lesion tissues of patients with OLP (Figure 4D). RT‐qPCR was conducted to verify the mRNA expression of NFIA in LPS‐treated HaCaT cells, and the expression level of NFIA was reduced in the LPS group compared with that in the control group (Figure 4E). HaCaT cells were infected with an overexpression lentiviral vector of NFIA. The mRNA expression of NFIA was upregulated in HaCaT cells of the OE‐NFIA group, and notably, the expression of S100A7 was downregulated (Figure 4F). A dual‐luciferase assay revealed that luciferase activity was reduced in the OE‐NFIA group compared to that in the OE‐NC group, indicating that the overexpression of NFIA suppressed the transcriptional activity of S100A7 (Figure 4G). ChIP was used to detect the enrichment of the S100A7 promoter region by anti‐NFIA, and a significant increase in the enrichment of the S100A7 promoter was found in the OE‐NFIA group relative to the OE‐NC group (Figure 4H).

### 3.5 The Anti‐Apoptotic and Anti‐Inflammatory Properties of OE‐NFIA Were Abated by Overexpression of S100A7

**FIGURE 5:**
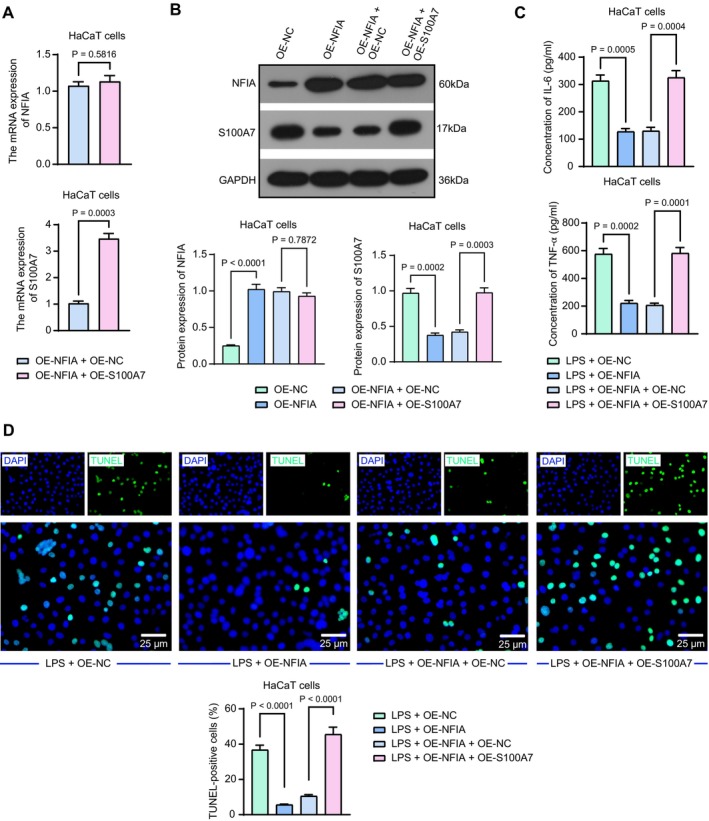
The inhibitory effects of NFIA on inflammatory response and apoptosis in HaCaT cells are reversed by S100A7 overexpression. (a) The mRNA expression of NFIA and S100A7 in HaCaT cells infected with OE‐NFIA + OE‐NC or OE‐S100A7 was examined using RT‐qPCR. (b) The protein expression of NFIA and S100A7 in HaCaT cells infected with OE‐NC, OE‐NFIA, OE‐NFIA + OE‐NC, or OE‐S100A7 was examined using Western blot analysis. (c) IL‐6 and TNF‐α levels in HaCaT cells after infection were examined using ELISA. (d) The representative images and TUNEL‐positive cells in HaCaT cells after infection. *p* values were analyzed by unpaired *t*‐tests or one‐way ANOVA. Data are the means ± SEM and are representative of three independent experiments.

HaCaT cells were first infected with the lentiviral vectors OE‐NC, OE‐NFIA, OE‐NFIA + OE‐NC, and OE‐NFIA + OE‐S100A7. RT‐qPCR was performed to detect the expression of NFIA and S100A7 in HaCaT cells. There was no significant change in the mRNA expression of NFIA, but the mRNA expression of S100A7 was significantly elevated in the OE‐NFIA + OE‐S100A7 group relative to the OE‐NFIA + OE‐NC group (Figure 5A). The protein expression of NFIA and S100A7 in HaCaT cells was investigated using western blot analysis (Figure 5B). The protein expression of NFIA was elevated, and that of S100A7 was decreased in the OE‐NFIA group compared to that in the OE‐NC group. Consistent with the RT‐qPCR results, OE‐S100A7 only induced the expression of S100A7 without altering NFIA. Under LPS exposure, ELISA for the secretion of IL‐6 and TNF‐α in HaCaT cells revealed that IL‐6 and TNF‐α levels were significantly lower in the OE‐NFIA group, and IL‐6 and TNF‐α secretion was enhanced by OE‐S100A7 (Figure 5C). The apoptosis rate of HaCaT cells was detected using the TUNEL assay. Apoptosis was significantly reduced in the OE‐NFIA group compared to that in the OE‐NC group, which was reversed by OE‐S100A7 (Figure 5D).

### 3.6 NFIA/S100A7 Axis Is Involved in OLP Progression

**FIGURE 6:**
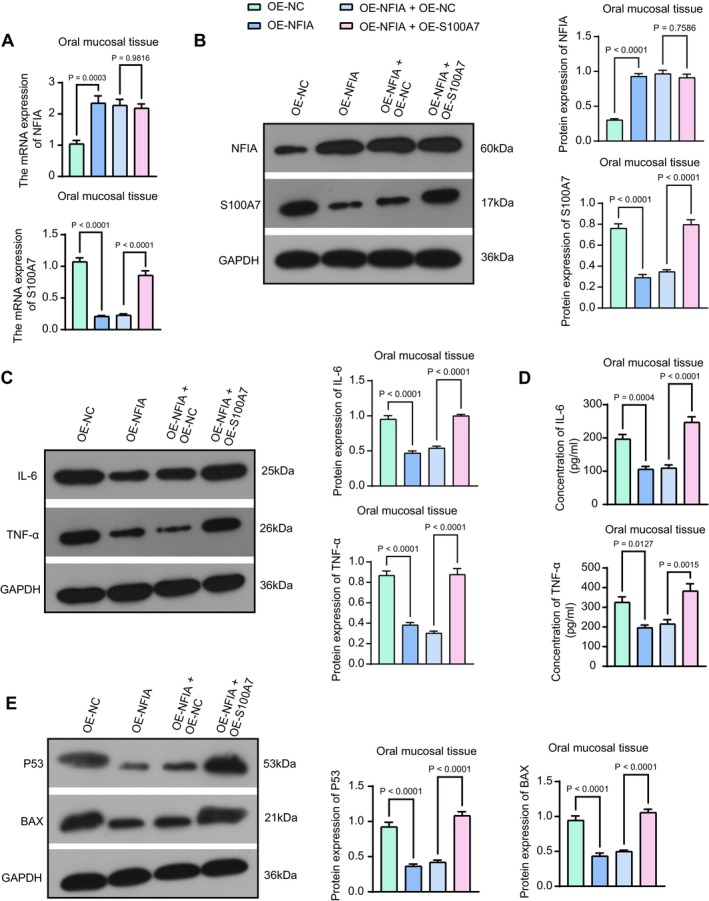
NFIA/S100A7 axis is involved in OLP in vivo. (a) The mRNA expression of NFIA and S100A7 in lesion tissues of mice was assessed using RT‐qPCR. (b) The protein expression of NFIA and S100A7 in the lesion tissues of mice was examined using Western blot analysis. (c) The protein expression of IL‐6 and TNF‐α in the lesion tissues of mice with different treatments was examined using western blot analysis. (d) IL‐6 and TNF‐α levels in the lesion tissues of mice with different treatments were examined using ELISA. (e) The protein expression of P53 and BAX in the lesion tissues of mice with different treatments was examined using western blot analysis. *p* values were analyzed by one‐way ANOVA. Data are the means ± SEM (*n* = 5).

To further verify that NFIA affects inflammation and apoptosis in OLP lesion tissues by regulating the transcriptional level of S100A7, we infected mice with lentiviral vectors OE‐NC, OE‐NFIA, OE‐NFIA + OE‐NC, and OE‐NFIA + OE‐S100A7, followed by OLP modeling. The results of RT‐qPCR (Figure 6A) and western blot analysis (Figure 6B) were consistent in that OE‐NFIA enhanced its expression and repressed that of S100A7, whereas OE‐S100A7 exogenously increased its expression without affecting NFIA expression. Western blot analysis and ELISA showed that the inflammatory response was reduced in the OE‐NFIA group compared with that in the OE‐NC group, and the inflammatory response was more severe in the OE‐NFIA + OE‐S100A7 group (Figure 6C,D). The assessment of pro‐apoptotic markers showed that overexpression of NFIA repressed the protein expression of P53 and BAX (Figure 6E) in the lesion tissues, which was reversed by overexpression of S100A7.

## 4 Discussion

Clinically, six types of OLP, namely reticular, papular, plaque‐like, erosive, ulcerative, and bullous, have been identified [14]. Although reticular OLP is rather easy to control, erosive OLP is tremendously painful and refractory to therapies, restraining the quality of life of patients [15, 16]. The present study investigated the role of S100A7 in OLP and showed that S100A7 knockdown reduced the keratinocyte inflammatory response and apoptosis in vitro and in vivo. Downregulation of the transcription factor NF1A caused the upregulation of S100A7 in keratinocytes, thereby inducing keratinocyte dysfunction in OLP. Our study provides novel insights into the molecular mechanisms by which NF1A/S100A7 regulates the development of OLP.

S100A7 is an EF‐hand type calcium‐binding protein with a molecular weight of 11.4 kDa, localized in the cytoplasm of keratinocytes, and distributed at the cell periphery in terminally differentiated keratinocytes [17]. Stolte et al. recently reported that S100A7 (*p* < 0.001) and TNFα (*p* < 0.001) mRNA levels were significantly upregulated in OLP tissue compared to healthy tissue, and clinical severity (25.21 ± 9.77) of OLP correlated positively with oral health‐related quality of life and S100A7 expression (*p* = 0.402) [18], which was consistent with our prediction and experimental results. In addition, S100A7 has been identified as a major contributing factor in the occurrence of oral cancer and induces local tumor progression [19], indicating its role in promoting malignant transformation. Although the specific function of S100A7 in OLP has not been fully revealed, Bielecka et al. showed that the effects of ursolic acid on psoriasis‐associated inflammation were related to its limitation on IL‐6 production in response to inflammatory stimuli and the expression of psoriatic biomarker S100A7 [20]. Moreover, human S100A7 induces mature IL‐1α expression in normal human epidermal keratinocytes, which is dependent on RAGE‐p38 MAPK and calpain‐1 [21]. Immune dysregulation may play a vital role in the pathogenesis of OLP, especially the overproduction of inflammatory mediators [22], and overexpression of various pro‐inflammatory factors, including IL‐6 and TNF‐α, has been found in OLP lesions, peripheral blood, and saliva [23]. Considering that OLP is characterized by massive cell apoptosis in the keratinocytes of the oral mucosa [24, 25], we assessed the function of S100A7 in modulating keratinocyte apoptosis in addition to its well‐known role in the inflammatory response. As expected, sh‐S100A7 exerted anti‐inflammatory and anti‐apoptotic effects in vitro and in vivo.

Under the condition of hidradenitis suppurativa, an inflammatory skin disease, the disruption of the S100A enhancer or the pharmacological inhibition of transcription factor IRF3 efficiently reduced the production of inflammatory regulators [26], indicating the pro‐inflammatory properties of S100A7 might be related to its upstream transcription factor. Moreover, Hiraike et al. reported that NFIA downregulated pro‐inflammatory cytokines in adipocytes to ameliorate adipose tissue inflammation by binding to the regulatory region of the Ccl2 gene and downregulating its transcription [27]. Here, we identified the binding relationship between NF1A and the S100A7 promoter in HaCaT cells. Overexpression of NFIA decreased the circulation of inflammatory cytokines, including IL‐6 and TNF‐α, and promoted regression of atherosclerosis in apolipoprotein E‐deficient mice, possibly through the nuclear factor kappa B (NF‐κB) pathway [28]. Nonetheless, to the best of our knowledge, its function in regulating keratinocyte apoptosis has not yet been revealed, which highlights the novelty of our study.

Given the fact that the NF‐κB signaling has been widely reported to be involved in LPS‐induced keratinocyte inflammation and apoptosis [29] and that S100A7 regulates the NF‐κB signaling under different inflammatory conditions [30], we posited that the NF‐κB signaling might be a downstream pathway of the NF1A/S100A7 pathway in keratinocytes, which needs to be validated in our following research. Sumardika et al. showed that the binding of S100A8/A9 to neuroplastin‐β mediates the activation of NFIA, which is linked to anchorage‐independent growth, motility, and invasiveness [31]. This paradoxical mechanism is yet to be elucidated and may be partly attributed to different microenvironments. Finally, as another transcription factor of S100A7, CREB1 was found to be involved in the dermal toxicity of alternariol in mice [32]. We cannot exclude the possibility that CREB1 also influences S100A7 in the context of OLP.

The presented study focused on reticular samples in the in vivo model, which poses a significant limitation. The use of multiple types of pathology samples for animal modeling could indeed improve the importance of the findings and provide further validation. Such studies are planned as a follow‐up analysis.

## 5 Conclusion

In conclusion, we present evidence that NF1A downregulation in keratinocytes exacerbates apoptosis and inflammatory responses by promoting the expression of S100A7. Targeting NF1A/S100A7 could be an effective strategy for OLP management.

## Conflicts of Interest

The authors declare no conflicts of interest.

## Supporting information


**Data S1.** kjm270052‐sup‐0001‐supinfo.docx.

## Data Availability

The data that support the findings of this study are available on request from the corresponding author. The data are not publicly available due to privacy or ethical restrictions.
