# Supplementary material for: Deletion of NFIA Leads to Activation of S100A7 and Inflammatory Response‐Induced Apoptosis of Keratinocytes in Oral Lichen Planus Progression
Source: Kaohsiung J Med Sci. 2025 Jun 25;41(9):e70052. doi: 10.1002/kjm2.70052 (PMC12412573; doi:10.1002/kjm2.70052)
Supplement: Supplementary file 1 — Data S1. kjm270052‐sup‐0001‐supinfo.docx. [file KJM2-41-e70052-s001.docx]

**Supplementary Material 1** Representative oral examination images of patients with reticular, papular, and erosive OLP

**Reticular**

**
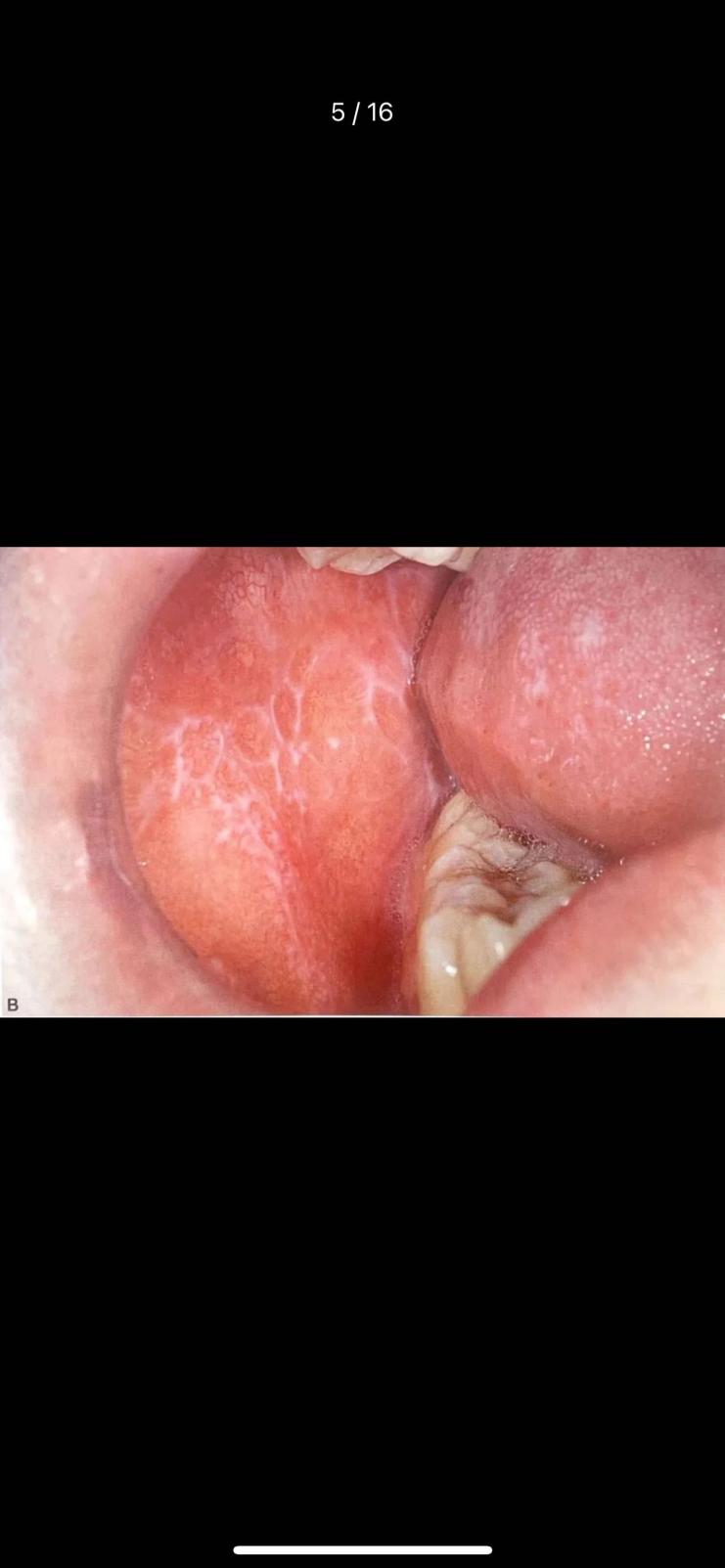
**

**Papular**

**
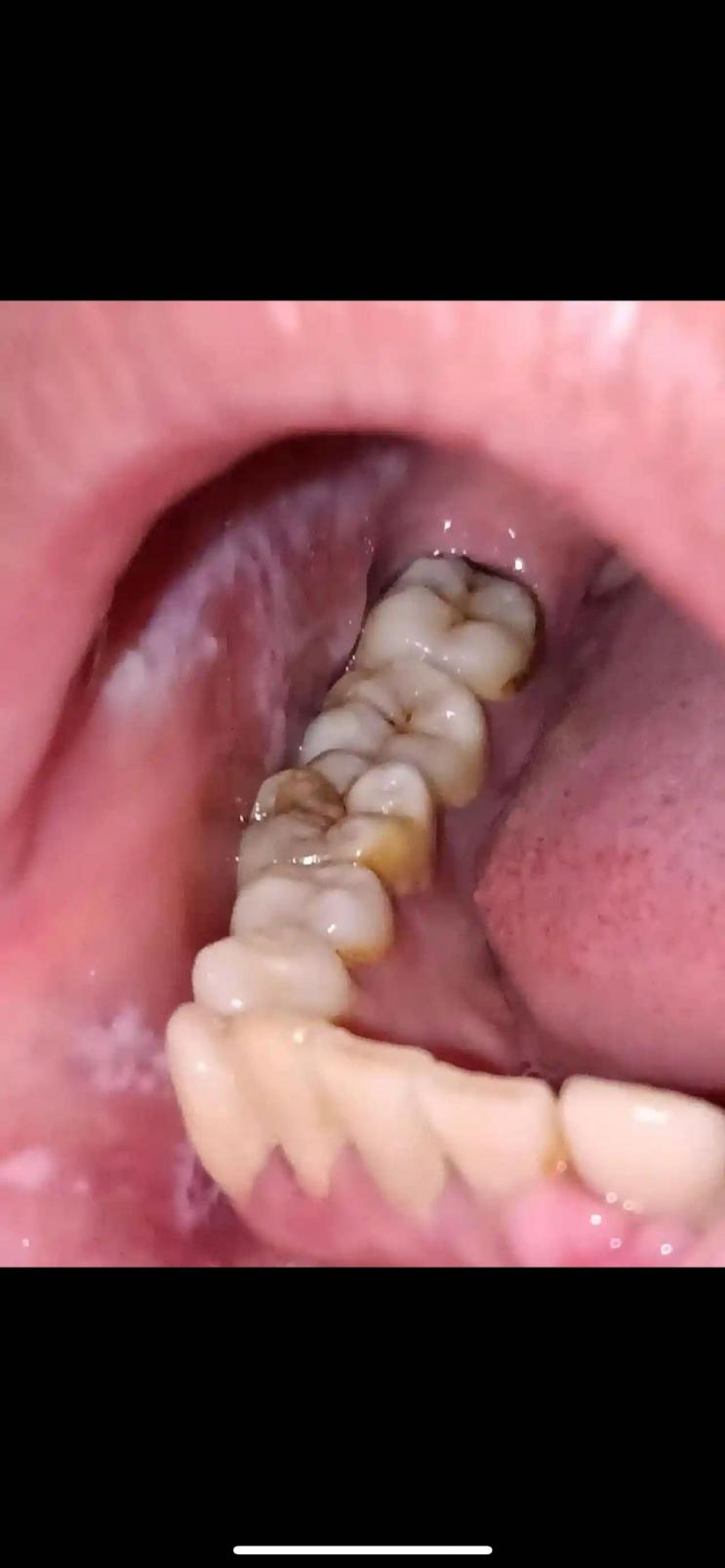
**

**Erosive**

**
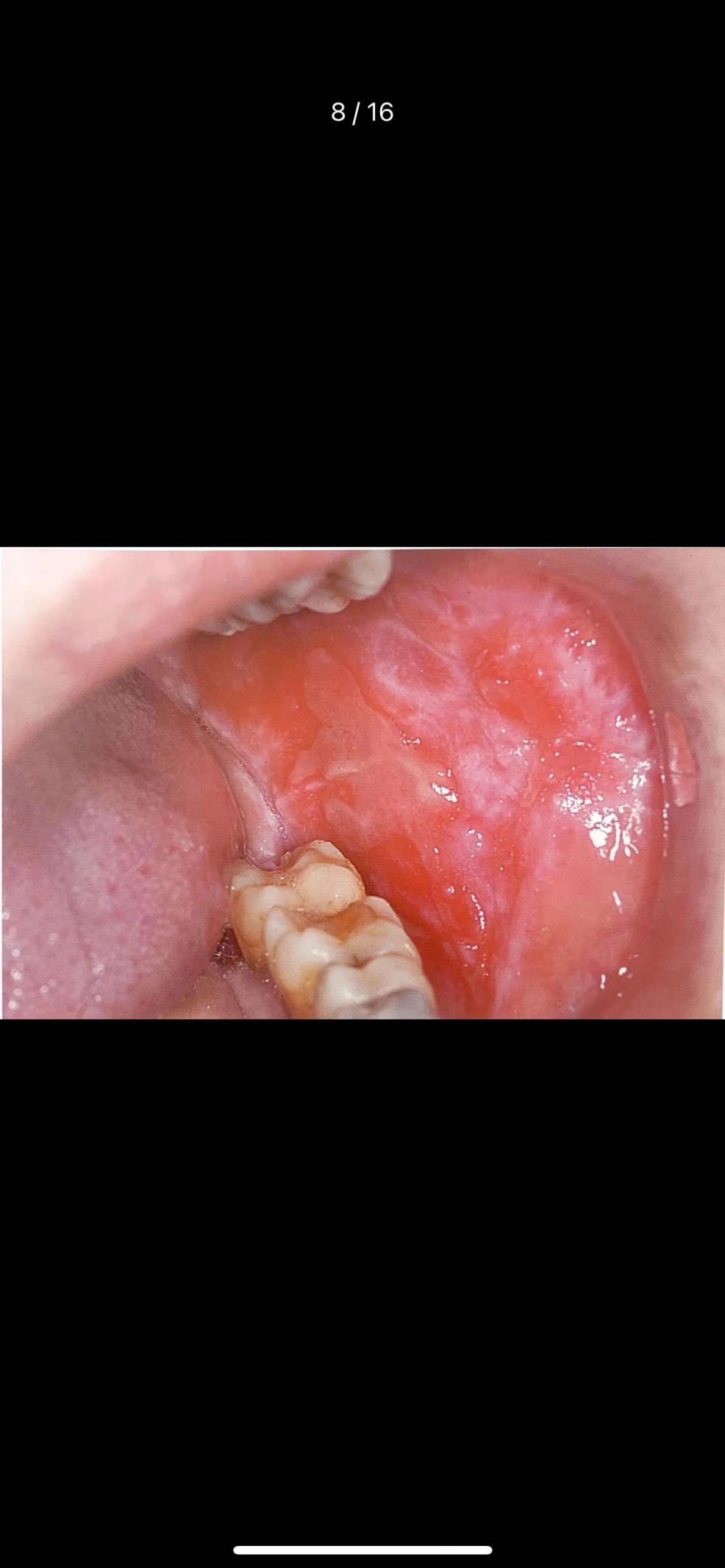
**
